# Data mining in conservation research using Latin and vernacular species names

**DOI:** 10.7717/peerj.2202

**Published:** 2016-07-19

**Authors:** Ivan Jarić, Franck Courchamp, Jörn Gessner, David L. Roberts

**Affiliations:** 1 Department of Biology and Ecology of Fishes, Leibniz-Institute of Freshwater Ecology and Inland Fisheries, Berlin, Germany; 2 Institute for Multidisciplinary Research, University of Belgrade, Belgrade, Serbia; 3 Ecologie, Systématique, and Evolution, Univ. Paris-Sud, CNRS, AgroParisTech, Université Paris Sud (Paris XI), Orsay, France; 4 Durrell Institute of Conservation and Ecology, School of Anthropology & Conservation, Marlowe Building, University of Kent, Canterbury, Kent, United Kingdom

**Keywords:** Internet, Common name, Social network, Vernacular name, Data mining, Scientific name, Latin name

## Abstract

In conservation science, assessments of trends and priorities for actions often focus on species as the management unit. Studies on species coverage in online media are commonly conducted by using species vernacular names. However, the use of species vernacular names for web-based data search is problematic due to the high risk of mismatches in results. While the use of Latin names may produce more consistent results, it is uncertain whether a search using Latin names will produce unbiased results as compared to vernacular names. We assessed the potential of Latin names to be used as an alternative to vernacular names for the data mining within the field of conservation science. By using Latin and vernacular names, we searched for species from four species groups: diurnal birds of prey, Carnivora, Primates and marine mammals. We assessed the relationship of the results obtained within different online sources, such as Internet pages, newspapers and social media networks. Results indicated that the search results based on Latin and vernacular names were highly correlated, and confirmed that one may be used as an alternative for the other. We also demonstrated the potential of the number of images posted on the Internet to be used as an indication of the public attention towards different species.

## Introduction

Analyses of the Internet provide a rich source of information and contribute considerably to conservation activities and evaluation (Wilson et al., 2007; Kim et al., 2014). Internet search engines, web-based data assessments, and social network mining approaches are increasingly used to assess public awareness regarding nature conservation (Funk & Rusowsky, 2014; Arts, Van der Wal & Adams, 2015). Internet-based research reduces costs and time, while at the same time it avoids some of the problems commonly encountered in large physical surveys, such as systematic sampling bias and a lack of insight in temporal trends (Edwards et al., 2013; Do et al., 2014; Kim et al., 2014).

In conservation science, assessments of trends and priorities often focus on species as the management unit (e.g., Wilson et al., 2007; Sitas, Baillie & Isaac, 2009; Muter et al., 2013; Żmihorski et al., 2013; Kim et al., 2014; Roberge, 2014). Given that the online media represent one of the major routes through which information related to conservation issues reaches decision makers, interest groups and the public, it is considered that the media coverage intensity has a direct influence on public opinion (Barua, 2010; Jacobson et al., 2012; Proulx, Massicotte & Pépino, 2014; Bombaci et al., 2015). Thus, it is assumed that species coverage by online media indicates public perception of conservation issues and potential biases in public interest, and represents popularity of a species and its public appeal (Sitas, Baillie & Isaac, 2009; Żmihorski et al., 2013; Roberge, 2014). At the same time, species coverage in scientific publications and databases is considered to represent available knowledge and scientific attention (Sitas, Baillie & Isaac, 2009; Jarić, Knežević-Jarić & Gessner, 2015; Fleming & Bateman, in press). Data mining within different databases and the Internet in general based on species names is increasingly recognized as a valuable tool in conservation research.

Studies on species coverage in online media commonly focus on Internet pages, newspaper articles, social media networks and different Internet search engines, while the search is commonly conducted by using species vernacular names (Barua, 2010; Jacobson et al., 2012; Bhatia et al., 2013; Muter et al., 2013; Żmihorski et al., 2013; Kim et al., 2014; Roberge, 2014). However, use of species vernacular names for web-based data search is problematic. Firstly, numerous different names for the same species exist in different languages. Secondly, even within the same language there can be multiple vernacular names for the same species. Thirdly, some vernacular names are used for multiple species or are vague, for example wolf, lynx, elephant, or imperial eagle. Fourthly, some vernacular names are often used in ways that are not specific to species, for example company names, articles dealing with sport teams (often named after a charismatic animal), machines and military equipment, toponyms (names of mountains, rivers, settlements), and personal names. In addition, species from certain groups, such as amphibians and insects, may also lack English vernacular names (Sitas, Baillie & Isaac, 2009). Use of vernacular names in bibliographic analysis therefore has the potential to lead to biased results, or at least a considerable amount of effort will be expended identifying the relevance of research results.

Latin names, on the other hand, have the advantage of being universally used, irrespective of language, and overlaps in names among species are comparably less frequent. Nevertheless, they have been only rarely used for data-mining in scientific studies (e.g., Wilson et al., 2007; Sitas, Baillie & Isaac, 2009), probably because it is unclear whether a search using Latin names will produce unbiased results as compared to vernacular names, as one may expect them to be used predominantly in the scientific community. For example, press articles using Latin names are expected to be rare. To our knowledge, this issue has yet to be addressed.

Here we assessed the potential use of Latin names as an alternative to vernacular names for data mining within the field of conservation science. We assessed the relationship of search results based on Latin and vernacular names of species from different species groups. This relationship was assessed within different online sources, such as Internet pages, newspapers, social media networks and images posted on the Internet.

## Methods

Species lists, with their Latin and vernacular names (specifically English common names), were obtained from the IUCN Red List database (IUCN, 2015). We focused on four charismatic and endangered animal groups: diurnal birds of prey (i.e., members of the order Accipitriformes, Falconiformes and Cathartiiformes), Carnivora, Primates and marine mammals (i.e., cetaceans and pinnipeds). Within each of the four species groups, 20 species were selected for the analyses by stratified random sampling, namely by sampling randomly within two subgroups that comprise species receiving high research attention and those of low scientific focus. Research focus was defined as the number of scientific publications per species (Jarić, Knežević-Jarić & Gessner, 2015; I Jarić et al., 2015, unpublished data). This was conducted in order to ensure that both charismatic and neglected species were included in the sample. During the sampling, only the species with vernacular names not likely to produce mismatches and those without overlapping names were selected for the analysis. The exclusion of species that are likely to produce substantial amount of mismatches in the results may potentially represent a source of a bias. Nevertheless, a reliable assessment of the media coverage regarding such species would not have been possible.

Assessment of media coverage for each of the selected species was carried out within five different sources. The number of Internet pages containing the name of each species was estimated using the Google search engine, and the presence of each species was also evaluated within each of the two major social networks, Twitter and Facebook, as well as within the websites of selected major newspapers from different countries—The New York Times, The Guardian, Le Monde, Washington Post, and Asahi Shimbun (i.e., analyzed together). Furthermore, the number of pictures posted on the Internet on each of the studied species was also estimated, as an indication of public interest and species appeal. Search within the two assessed social networks, newspapers and pictures was conducted through the Google search engine, with the following search queries: (1) Twitter –[“*species name*” site:twitter.com]; (2) Facebook –[“*species name*” site:facebook.com]; (3) Newspapers –[“*species name*” (site:nytimes.com OR site:theguardian.com OR site:lemonde.fr OR site:washingtonpost.com OR site:asahi.com)]; (4) Photographs –[“*species name*” (filetype:png OR filetype:jpg OR filetype:jpeg OR filetype:bmp OR filetype:gif OR filetype:tif OR filetype:tiff)]. For species with recognized vernacular synonyms (IUCN, 2015), all names listed in their IUCN Red List assessments were used to conduct the search. Since there were no date ranges established for collected data, all results dating prior to the time of the assessment (in December 2015) were included in the analysis.

Statistical analyses were conducted using the R programming language (R v.3.0.2). For R scripts see Supplemental Information 2 (also available in the online repository, https://dx.doi.org/10.6084/m9.figshare.3381073.v2). Since the variables were not normally distributed (Lilliefors (Kolmogorov–Smirnov) test, *p* < 0.001), nonparametric tests were applied. Relationship between the search results based on vernacular and Latin species names, within each of the four studied species groups and the five search types applied, was assessed using a Spearman’s Rank test.

## Results

**Figure 1:**
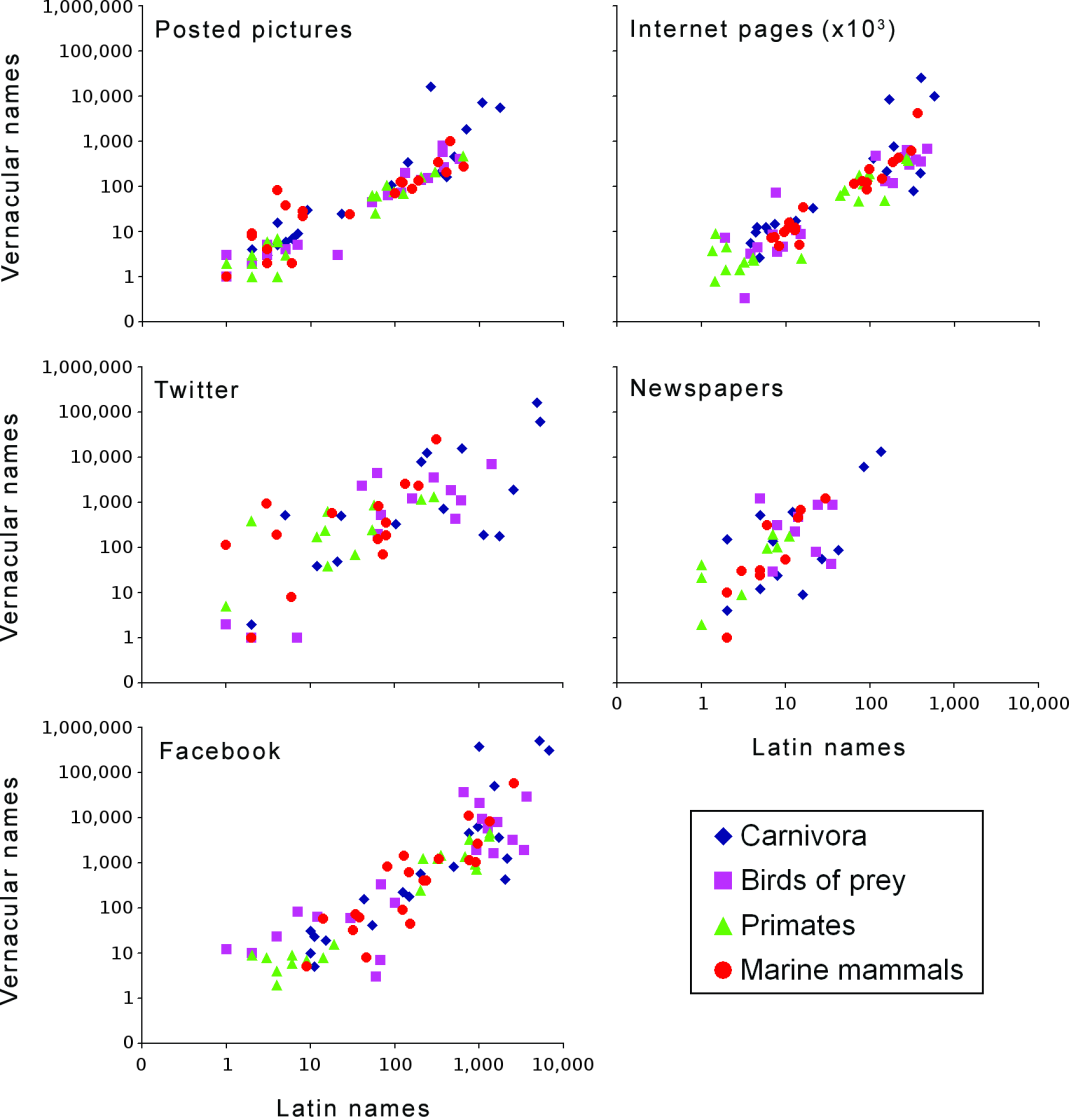
Relationship between search results per species based on vernacular and Latin species names, conducted within the four studied species groups and the five assessed sources; axes represent logarithmic scales. Presented data were transformed using *x*←*x* + 1, in order to allow presentation in log-plots of results with the value of zero; for the original dataset, see Supplemental Information 1 (also available in the online repository, https://dx.doi.org/10.6084/m9.figshare.3381073.v2).

All of the searched sources produced more results when vernacular names were used, especially newspapers (Fig. 1). Average proportion between the coverage for Latin and vernacular was the lowest for newspaper articles (0.04) and highest for posted pictures (0.85).

The results indicated strong positive correlations between the number of search results based on Latin and vernacular names, in each of the four assessed species groups and for each of the five used search types (Fig. 1 and Table 1). The strongest correlations were observed for posted pictures, followed by Internet pages.

**Table 1:** Coefficients of correlation between search results based on vernacular and Latin species names, conducted within the four studied species groups and the five assessed search types (Spearman’s non-parametric correlation test, *p* < 0.01 for all assessed groups).

	Internet pages	Twitter	Facebook	Newspapers	Pictures
Birds of prey	0.854	0.738	0.773	0.833	0.905
Carnivora	0.889	0.835	0.880	0.851	0.919
Marine mammals	0.930	0.790	0.836	0.817	0.900
Primates	0.824	0.799	0.856	0.836	0.916

## Discussion

Results indicated that searches based on Latin and vernacular names were highly correlated, and confirmed that one can be used as a general proxy for the other. There are however three caveats that should be emphasized. Firstly, the results are only applicable when looking at quantitative data, such as the amount of coverage of different species in online media, since the similarity of the actual media content obtained by the two assessed search types was not the object of this study. Secondly, use of the Latin names may be less adequate in studies that analyse Internet search behaviour, for instance by using the Google Trends search engine, as the number of searches based on Latin names may be insufficient to obtain data (Kim et al., 2014). Lastly, very low species coverage in newspapers based on Latin names, as compared to vernacular names, indicates that Latin names are only rarely used by journalists. Consequently, assessments of species coverage within newspaper articles based on Latin names should be interpreted with due caution.

We assessed the two search methods within the five different online media categories that are commonly used by the scientific community for data mining. Twitter and Facebook currently represent the two most popular social networks and powerful research tools (Miller, 2011; Naaman, Becker & Gravano, 2011; Roberge, 2014; Papworth et al., 2015). News media are a common venue for broadcasting science topics to the general public, which makes them suitable to reflect public attention and popular attitudes (Muter et al., 2013; Veríssimo et al., 2014; Papworth et al., 2015). Besides the online media types that are commonly assessed by the scientific community, we also demonstrated the use of the number of images posted on the Internet as an indication of the public attention towards different species. While the value of image sharing through social networking sites as a data source was recognized by previous studies (Barve, 2014), to our knowledge this is the first illustration of the use of web-based images as a search tool within conservation science. Suitability of this method for data mining was also indicated by high similarity in the coverage of posted images based on Latin and vernacular names (Fig. 1), especially when compared with the coverage within other sources.

Although the assessment was focused only on species with vernacular names that were not likely to produce mismatches, they were nevertheless still observed during the analysis. At the same time, results based on the Latin names were much more consistent, which indicates better reliability of their use. Search within social networks was especially problematic, as many social network users assigned some species common names as personal usernames (Latin names seem to be rarely used as usernames), so any post or tweet made by such person will be recognized as a matching result for that species. It is nevertheless possible that the use of different and more detailed search criteria than those presented here could resolve this problem to an extent. Problems encountered by the appearance of unrelated results, produced by online media search with species vernacular names, were also recognized by other authors. In a study on media coverage of Florida panther (*Puma concolor coryi*), Jacobson et al. (2012) detected mismatches based on vernacular name search, such as sport team names. Presence of irrelevant material within newspaper articles obtained by searching with a vernacular species name was also observed by Barua (2010). In a study by Roberge (2014), Twitter results based on the vernacular names search contained various mismatches such as names of sports teams, trademarks or product names, artist or character names, metaphors, and place names.

Many species are referred to in media by multiple vernacular synonyms and spelling variants. However, as stated by Aksnes & Browman (2016), practice of using only the most well-known vernacular name to conduct search is a potential source of bias, and may make such species underrepresented in results. Assessment of vernacular names based on a single language in the regions where multiple languages are used is also problematic, given that processes occurring in different media outlets may differ substantially (Bhatia et al., 2013). The use of English search phrases does not necessarily reflect worldwide patterns and might be therefore misleading (Funk & Rusowsky, 2014). This is especially important when bearing in mind that the areas of high species diversity are often characterized by a high level of linguistic diversity (Gorenflo et al., 2012).

As stated by Wilson et al. (2007), the species Latin names are the same in every language, and they are widely used by non-scientists. It is important to emphasize, however, that due caution is needed when using Latin names for data-mining purposes, due to their instability over time (Lepage, Vaidya & Guralnick, 2014). For instance, substantial numbers of species of birds of prey assessed in the current study are disputed regarding their taxonomic status or nomenclature. Our intention was to use the assessed species groups merely as an illustration of the presented method and to address the question we discussed here. In addition to the IUCN Red List database, other relevant databases and sources such as Avibase (Lepage, 2016) should be also consulted to resolve the status of the assessed species.

Our results indicated that Latin names may be used as an alternative to vernacular names. Nevertheless, it is important to note that the results presented here support search by Latin names only for assessments of the relative species coverage in media, i.e., either in time or when comparing among different species or species groups, since the use of absolute results could lead to large underestimations of the actual coverage.

##  Supplemental Information

10.7717/peerj.2202/supp-1 Supplemental Information 1 Dataset Search results based on vernacular and Latin species names, conducted within the four studied species groups and the five assessed online sources

10.7717/peerj.2202/supp-2 Supplemental Information 2 Scripts for statistical analysis Scripts for statistical analysis (Lilliefors normality test, Spearman’s non-parametric correlation test), made using the R programming language (R v.3.0.2).

## Additional Information and Declarations

### Data Availability

The following information was supplied regarding data availability: Figshare: https://dx.doi.org/10.6084/m9.figshare.3381073.v2.
